# Prolactin Relationship with Fertility and In Vitro Fertilization Outcomes—A Review of the Literature

**DOI:** 10.3390/ph16010122

**Published:** 2023-01-13

**Authors:** Mirela E. Iancu, Alice I. Albu, Dragoș N. Albu

**Affiliations:** 1 Endocrinology Department, ARTHOPE SRL, 077190 Bucharest, Romania; 2 Endocrinology Department, Elias Emergency Hospital, 011461 Bucharest, Romania; 3 Endocrinology, 2nd Clinical Department, General Medicine Faculty, ‘Carol Davila’ University of Medicine and Pharmacy, 050454 Bucharest, Romania; 4 Obstetrics and Gynecology, 2nd Clinical Department, Dentistry Faculty, ‘Carol Davila’ University of Medicine and Pharmacy, 050454 Bucharest, Romania

**Keywords:** prolactin, in vitro fertilization, hyperprolactinemia, transient hyperprolactinemia, pregnancy rate

## Abstract

Hyperprolactinemia is a known cause of amenorrhea and infertility. However, there is an increasing body of evidence suggesting that prolactin is involved in multiple physiological aspects of normal reproduction. Thus, the present paper aims to review the current literature regarding the relationship between serum prolactin level and in vitro fertilization (IVF)/intracytoplasmic sperm injection outcome and the role of dopamine agonists treatment in IVF success. Moreover, the mechanisms by which prolactin may exert its role in fertility and infertility were summarized. Although not all studies agree, the available evidence suggests that higher prolactin levels in follicular fluid are associated with increased oocytes competence, but also with positive effects on corpus luteum formation and survival, endometrial receptivity, blastocyst implantation potential and survival of low-motile sperm. Transient hyperprolactinemia found in IVF cycles was reported in most of the studies not to be related to IVF outcome, although a few reports suggested that it may be associated with higher implantation and pregnancy rates, and better-cumulated pregnancy outcomes. Administration of dopamine agonists for hyperprolactinemia preceding IVF treatment does not seem to negatively impact the IVF results, while treatment of transient hyperprolactinemia during IVF might be beneficial in terms of fertilization rates and conception rates. Due to limited available evidence, future studies are necessary to clarify the optimal level of circulating prolactin in patients performing IVF and the role of dopamine agonist treatment.

## 1. Introduction

An increasing body of evidence supports the pleiotropic role of prolactin in reproduction, growth, metabolism, electrolyte transport, behaviour, immunity and carcinogenesis [1]. Regarding the physiological effects, aside from the traditional role on lactation, several studies have suggested a more complex role of prolactin in reproduction by its benefic effects on oocytes development [2,3,4,5], corpus luteum formation and survival [6], implantation [7], steroidogenesis [8,9] and immunomodulatory effects [10,11]. Thus, a certain level of circulating prolactin might be necessary for optimal reproductive outcomes. During the menstrual cycle, fluctuations of the prolactin level were noticed, supporting the involvement of prolactin in variate aspects of reproduction. During controlled ovarian stimulation (COS) for assisted reproductive technology a particular dynamic of serum prolactin level, characterized by transient hyperprolactinemia, was also reported [8,12,13] with incompletely known significance and impact on fertility treatment outcome.

Regarding its pathophysiological importance, the most studied implication is hyperprolactinemia-induced infertility. The main mechanism by which hyperprolactinemia leads to infertility is a decrease in gonadotrophin-releasing hormone (GnRH) secretion with subsequent hypogonadotropic hypogonadism and anovulatory infertility. Experimental studies suggest that high levels of prolactin might additionally affect fertility by alteration in endometrial function and implantation through both structural and immunological factors [14]. Whether these effects on endometrium are significant in humans is debated, being suggested that they have only marginal significance [14,15].

Although the exact prevalence of hyperprolactinemia in infertile women is not clearly established, it appears to be at least ten times higher than in the general population [16]. Moreover, females with tubal factor infertility also present more commonly with abnormal prolactin concentration, suggesting that hyperprolactinemia may further decrease fertility potential in this category of patients [17].

Thus, the question arises whether hyperprolactinemia should be treated before and during assisted reproduction techniques (ART) as these procedures may overcome the detrimental effects on ovulation induced by high prolactin levels [14]. On the other hand, treatment for hyperprolactinemia might interfere with the possible beneficial effects of prolactin on other aspects of reproductive function. However, no guidelines address the aspect of optimal prolactin levels during infertility treatment for optimal outcomes. Therefore, we aimed to review the existing literature regarding the relationship between serum prolactin level and in vitro fertilization/intracytoplasmic sperm injection (ICSI) outcome and the role of dopamine agonists treatment in in vitro fertilization (IVF) success. Moreover, the mechanisms by which prolactin may exert its role in fertility and infertility were summarized.

We performed a PubMed database search using the following terms “prolactin” and “in vitro fertilization” or “intracytoplasmic sperm injection”. We retrieved 258 results and we further perfected the search by analyzing the abstracts available for each of the studies and by searching for articles of interest included in the reference list of relevant articles. Finally, 88 studies were included in the current material.

## 2. The Mechanisms behind Reduced Fertility in Hyperprolactinemic Patients

**Figure 1:**
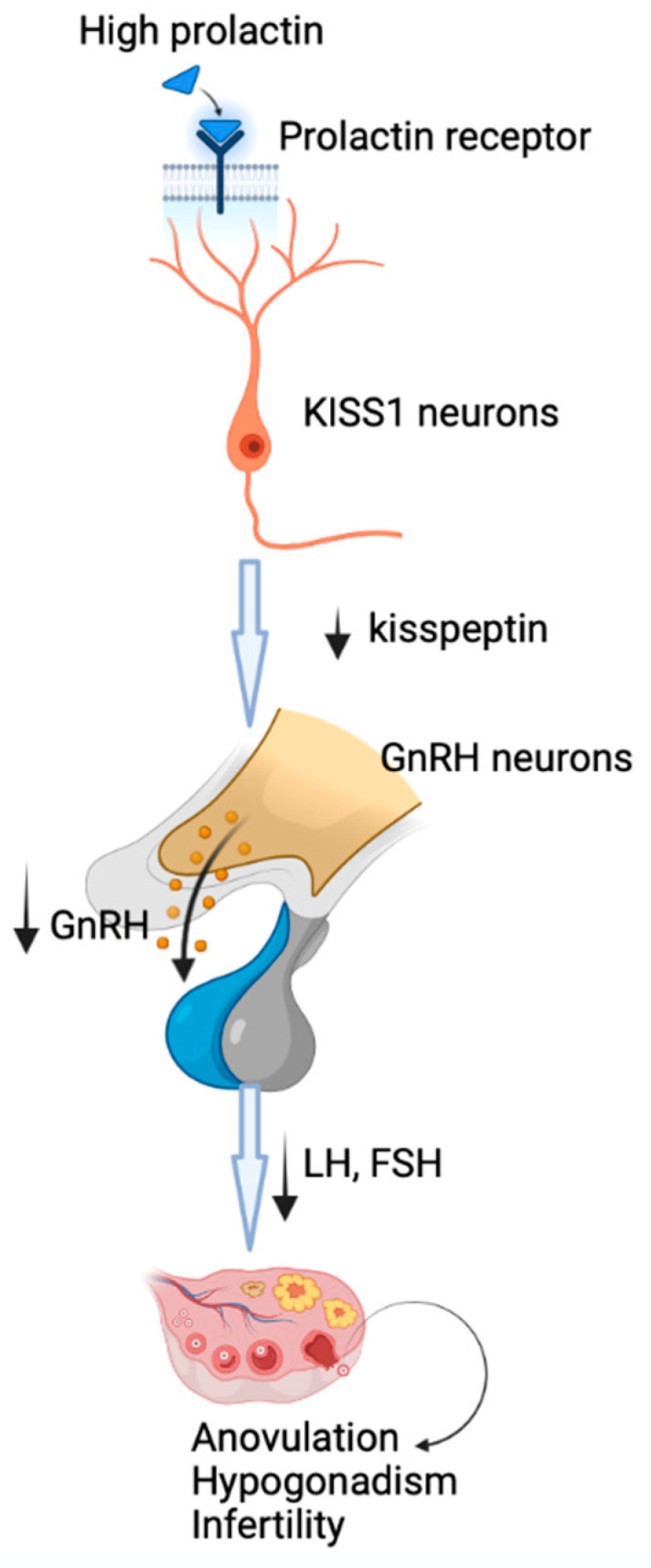
The mechanism of induction of hypogonadotropic hypogonadism by hyperprolactinemia. Prolactin acts through prolactin receptors on metastasis-suppressor kisspeptin-1 neurons, with downstream disruption of GnRH secretion, followed by decreased production of gonadotropins, hypogonadotropic hypogonadism and anovulation.

The main mechanism by which prolactin may lead to reproductive dysfunction is the inhibition of GnRH secretion, followed by decreased production of gonadotropins, hypogonadotropic hypogonadism and anovulation. New evidence has shown that the inhibition of hypothalamic secretion of GnRH involves metastasis-suppressor kisspeptin-1 neurons that express prolactin receptors [14] through which prolactin may exert its inhibitory effects at this level, with downstream disruption of GnRH secretion (Figure 1) [1]. The central role of kisspeptin-1 in mediating the prolactin action on GnRH secretion is highlighted by the restoration of fertility by kisspeptin-1 administration [1].

**Figure 2:**
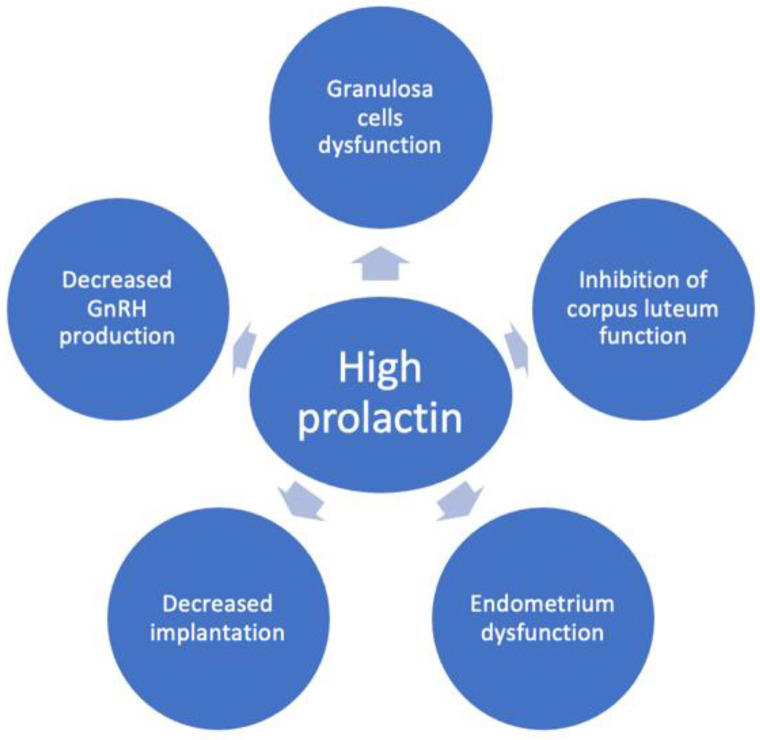
The mechanisms of reduced female fertility in hyperprolactinemia. Hyperprolactinemia may contribute to infertility by inducing decreased GnRH production, granulosa cell dysfunction, inhibition of corpus luteum function, endometrial dysfunction and decreased implantation.

Additionally, accumulating evidence indicates that prolactin also influences directly the granulosa cells’ function (Figure 2) [8], exerting a direct inhibitory effect on gonadotropin action in the ovary [9,18]. In granulosa cells, prolactin inhibits estradiol production [9,19,20,21] and stimulates progesterone production [20,22,23] by activating distinct signalling pathways [24]. The mechanism by which excess prolactin inhibits follicle-stimulating hormone (FSH)-induced estradiol secretion in preovulatory follicles has been reported to be due to the reduction of aromatase activity [20,22,25,26], whereas FSH-induced steroidogenic acute regulatory protein, P450 side-chain cleavage enzyme and 3β-hydroxysteroid dehydrogenase type 2 levels are amplified by prolactin [8]. The bone morphogenic protein system in growing follicles plays a key role in antagonizing prolactin receptor signalling actions in the ovary when exposed to high concentrations of prolactin [27].

Some authors found prolactin receptors only in the human luteinized granulosa cells, but not in early follicles, and concluded that prolactin may play a role in the mature follicle during the time of ovulation [28]. Moreover, clinical data support an inhibitory effect of high prolactin levels on the function of the corpus luteum (Figure 2) [29]. Thus, in hyperprolactinemic women with ovulatory cycles, it was shown that hyperprolactinemia leads to luteal phase insufficiency and low progesterone levels [29]. It was demonstrated that the short luteal phase precedes the full clinical picture of hyperprolactinemia, suggesting that luteal phase insufficiency may be the first sign accompanying an increase in prolactin levels or the consequence of less severe hyperprolactinemia [29]. Thus, mild hyperprolactinemia might be associated with an incomplete clinical picture, being more difficult to recognize. Some authors consider that hyperprolactinemic women with regular menstrual cycles, especially when COS is planned, are a problematic patient category to manage due to the lack of clear evidence-based recommendations [30]. Although macroprolactinemia may be the underlying cause of prolactin elevations in some of the cases, and screening for macroprolactinemia is suggested for every value > 25 ng/mL, it was suggested that macroprolactinemia cannot be considered a completely harmless condition as it may be associated with infertility and autoimmune conditions [30,31].

Experimental animal studies offer some insights into the additional mechanism by which high levels of prolactin might contribute to decreased fertility by alteration in endometrial function and implantation (Figure 2) [14]. Thus, metoclopramide-induced hyperprolactinemic mice were found to have fewer pinopodes in the endometrium and a lower pregnancy rate [32]. Moreover, the same mouse model has a different glycosaminoglycan content in the endometrium which might affect the decidualization process [33]. Another suggested mechanism is the immune dysregulation induced by high prolactin which may alter implantation and pregnancy (Figure 2) [10,12,34]. It is debated whether these effects on endometrium are significant in humans since several studies showed that ovulation induction in hyperprolactinemic women resulted in pregnancy in spite of persistently high prolactin levels [14,15].

## 3. Prolactin Roles in Fertility

Earlier studies demonstrated that follicular fluid prolactin is not synthesized locally but originates in the pituitary gland, reaches the ovary through passive diffusion from systemic circulation and is regulated by estrogen [35]. However, more recent data suggest that the ovary might be an extra-pituitary source of prolactin secretion [6]. Other extra-pituitary sites of the prolactin gene expression have been discovered, among which are the endometrium, T-lymphocytes, brain, skin, breast, follicular fluid, ovarian follicular cells and amniotic fluid. The preponderance of circulating prolactin originates from the pituitary and until now the precise role of extra-pituitary prolactin is unknown [30]. Three major prolactin isoforms have been detected in different biological fluids: monomeric, big prolactin and big, big prolactin (macroprolactin) [36]. Different prolactin isoforms have different functions [1]. The only prolactin encountered in follicular fluid in patients undergoing IVF was the small, potent isoform.

Taking into account the presence of prolactin in the follicular fluid and the expression of the prolactin gene in the ovarian granulosa cells, several studies evaluated prolactin’s influence on oocyte maturation with controversial results. Few studies reported a negative relationship between follicular fluid prolactin levels and oocyte competence. Thus, Lee et al. found higher prolactin concentrations in the follicular fluid of unfertilized oocytes [2] and Reinthaller et al. showed that advanced follicular maturation was associated with decreasing follicular fluid prolactin [3]. Other authors found no correlation between the follicular fluid prolactin level and IVF outcomes, challenging the role of monomeric prolactin in oocyte physiology and fertilization [36].

On the other hand, Lindner et al. demonstrated that oocyte maturation and capacity to be fertilized was predicted by higher prolactin contents in follicular fluid and contested possible adverse effects of prolactin on oocytes [4]. Similarly, Subramanian’s study attested the biological activity of follicular fluid prolactin and found that mature preovulatory follicles contained a significantly greater concentration of prolactin in comparison to immature follicles, but prolactin concentration did not correlate with ovum fertilizability [37]. Laufer et al. linked higher follicular fluid prolactin levels with an increased number of successful pregnancies, supporting the role of prolactin in oocyte maturation [38]. A small study of couples undergoing ICSI for male factor infertility found that prolactin, progesterone, growth hormone, interleukin-1 (IL-1) and tumour necrosis factor-α were higher in follicles yielding oocytes that subsequently fertilized compared with those of oocytes that failed to fertilize [39]. Furthermore, oocytes that underwent a more rapid development had higher levels of follicular LH, prolactin and IL-1 in contrast to those who only reached the 2-cell stage or 3-cell stage 40–44 h after ICSI [39].

**Figure 3:**
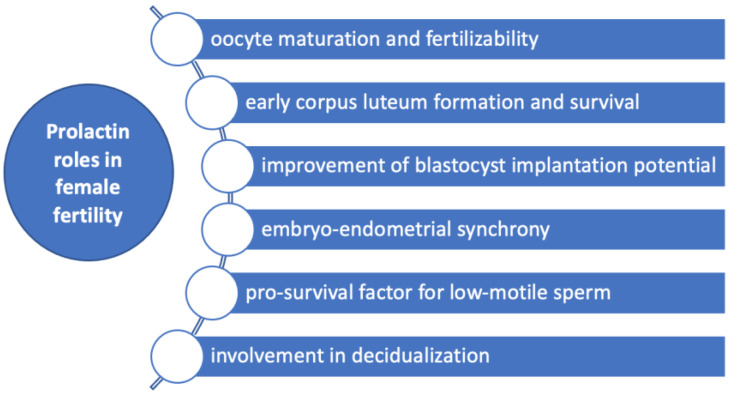
Prolactin roles in female fertility. Prolactin has several roles in female fertility by influencing oocytes maturation and fertilizability, early corpus luteum formation and survival, improvement of blastocyst implantation potential, decidualization, embryo-endometrial synchrony and acting as a pro-survival factor for low-motile sperm.

Experimental studies in bovine oocytes showed that both prolactin and growth hormone affect the morphology in metaphase II chromosomes in cumulus-enclosed oocytes in a biphasic dose-dependent manner [40], supporting a role for prolactin in oocyte maturation and developmental competence (Figure 3). Thus, prolactin in concentrations of 50 ng/mL decelerated the abnormal chromosome changes in cumulus-enclosed ageing bovine oocytes [40]. However, this effect was not found in denuded oocytes, suggesting that cumulus cells had a major role in mediating prolactin action probably by the prolactin receptors found in these cells [40,41]. In the opposite manner, concentrations 10–20 times higher than the normal range enhance destructive changes [40]. Animal studies in rats showed that prolactin administration may increase in vitro fertilization rate [42].

Prolactin may be also a significant contributor to early corpus luteum formation and survival through antiapoptotic actions on granulosa cells (Figure 3) [6]. These effects were demonstrated in luteinized granulosa cells due to prior exposure to human chorionic gonadotrophin (HCG), collected from IVF flush [6].

The implantation is the consequence of a complex interplay between the uterus and the blastocyst which are both active parts of this process. Temporal synchronization between blastocyst competency and uterine receptivity is an essential aspect of implantation that occurs in a limited time called ‘’window of implantation’’ and different factors have been demonstrated to interfere with this sensitive, tight-regulated process [6]. During the majority of ART protocols, the administration of hormones is performed in order to achieve an increased number of follicles within the ovary. However, these hormonal influences on the endometrium are drastically different from natural cycles and may alter the “window of implantation”, lowering the chances of achieving pregnancy irrespective of the embryo quality [43]. It was suggested that prolactin might be involved in the implantation by its ability to influence both the uterus and the blastocyst. Thus, it was shown that supplementation of culture medium with prolactin altogether with epithelial growth factor and 4-hydroxyestradiol improves IVF-derived mouse blastocyst implantation potential [6]. Decidualization is the process by which endometrial stromal cells undergo profound changes in preparation for the embryo to implant. Prolactin messenger ribonucleic acid (mRNA) was also found to be expressed in the late-luteal phase endometrium [44,45,46,47,48], being considered a marker of decidualization [49]. Repeated implantation failure (RIF) is a reproductive condition in which women are not able to achieve a pregnancy in at least three consecutive IVF cycles with the transfer of at least one good-quality embryo during each cycle. Some underlying causes have been proposed such as obesity, thrombophilia and autoimmunity, but in a large number of cases, the underlying defect cannot be pinpointed and is termed “unexplained RIF”. An embryo-endometrial asynchrony caused by premature expression of the decidualization marker prolactin during the luteal phase has been associated with RIF [50]. Moreover, decreased expression of prolactin was found in the endometrium of patients with RIF [51].

The interaction between sperm and biological components in the female reproductive tract may be an essential aspect of subfertility [52]. Thus, prolactin was studied among other components of the follicular fluid such as progesterone, myo-inositol and dopamine, regarding its ability to influence sperm parameters [52]. It was found that prolactin supplementation appears to act as a pro-survival factor by reducing reactive oxygen species in the case of low-motile sperm subpopulations [52]. Consequently, the authors concluded that treatment of subfertile semen samples with biological substances present in follicular fluid might be a new treatment strategy in IVF [52]. In regard to other aspects of the fertilization process, prolactin does not appear to have a role in sperm-zona pellucida binding or acrosome reaction [53].

## 4. The Relationship between Prolactin Levels and ART Outcomes

It was shown that prolactin levels are higher in stimulated than in spontaneous cycles [54]. Similar to natural cycles, there is a transient increase in serum prolactin in the late follicular phase of stimulated cycles [55], but a particular dynamic of prolactin levels in stimulated cycles was also reported, characterized by a transitory increase in prolactin levels. Thus, increases in prolactin levels were found at ovum pick up [13], before and after HCG administration [8,12], in the luteal phase [54], while other authors reported a bimodal serum prolactin profile with peaks 1 and 9 days after the maximal serum LH value [11]. It was shown that the increment on prolactin may reach a maximum level of 93.2 ng/mL after the ovum pickup procedure, but return to normal values before embryo transfer [13]. Moreover, patients undergoing laparoscopic surgery for oocyte retrieval during IVF procedures demonstrate significant, although transient elevations in prolactin levels, which are presumed to be caused by anaesthetic agents and surgical stress [56,57]. Thus, it was demonstrated that intraoperative prolactin levels increase significantly till mean values of 124 ng/mL, with a return to normal levels 48 h after surgery [58]. Other authors found that laparoscopy does not contribute significantly to prolactin rise, the COS being the real cause of prolactin increase [59].

Since prolactin is considered a stress hormone, it was suggested that prolactin increase during IVF treatment might be the consequence of stress. Thus, it was shown that infertile women might have a different personality profile in comparison with fertile women, characterized by more suspicion, guilt and hostility, accompanied by increased stress hormone levels such as prolactin and cortisol [60]. Some authors found that serum prolactin and cortisol levels increase during COS for IVF along with state anxiety score in infertile patients [59], while others found no association between psychological scores and stress hormones among which prolactin in patients performing IVF [61,62].

It seems that the variate IVF treatment regimens may have different effects on serum prolactin as reported by several studies. Thus, when comparing prolactin elevation in patients treated for IVF with Clomiphene citrate/HCG, clomiphene citrate/human menopausal gonadotrophin (HMG)/HCG or HMG/HCG, it was found that plasma prolactin concentrations were higher in HMG-treated group [63]. Furthermore, it was noticed that patients treated with HMG-HCG had lower prolactin concentrations when clomiphene citrate was associated in comparison with those treated with GnRH agonist (buserelin) for pituitary suppression [12]. In this study, only in the group treated with the GnRH agonist a significant rise in plasma prolactin was noticed before HCG administration [12]. While a marked prolactin peak after HCG injection was found in both groups, the elevation was more pronounced in the GnRH agonist group [12]. The same study showed that HCG administration is followed by prolactin elevation 48 h after injection in postmenopausal women and that surgical castration is associated with an increase in plasma prolactin which parallels the change in FSH concentration [12]. These data support that lactotroph cell secretion is stimulated by the human gonadotropins, independently from circulating estradiol levels [12]. In another study, the use of leuprolide acetate, a GnRH agonist, in association with human menopausal gonadotropins for oocytes retrieval was associated with higher serum estradiol and prolactin levels in comparison with patients receiving stimulation using only human menopausal gonadotropins [64]. The authors consider that this increase in prolactin level by GnRH and its agonistic analogues is due to the paracrine effects of the alpha and beta subunits of pituitary gonadotropins on lactotroph cells [64]. Women stimulated with GnRH agonist long protocol and HMG were found to have a significant increase in prolactin elevation compared with the patients treated with the short protocol, which was positively correlated with estradiol levels, but independent of midcycle LH surge [65]. Disagreeing, other authors support that LH surge might be involved in the midcycle [31] and luteal phase [11] rise of serum prolactin.

The involvement of increased estradiol levels in the occurrence of transient hyperprolactinemia is debated, with some authors confirming its contribution [66], and other authors founding no relationship between the two hormones [67].

Taking into account the negative impact of high prolactin levels on fertility and the complex involvement of prolactin in reproduction, several studies evaluated the relationship between transient hyperprolactinemia during COS and IVF outcomes. However, most of them, especially early studies, failed to demonstrate a relationship. Thus, Pattinson et al. showed that the percentage rise of prolactin after stimulation did not impact the peak estradiol levels achieved, the number of follicles seen, the number of eggs retrieved, or pregnancies obtained [55]. Similarly, in patients undergoing different ovarian stimulation protocols, Hummel et al. did not observe the injurious effects of transient hyperprolactinemia on the total number of oocytes, the number of mature oocytes, fertilization rates, cleavage rates, and pregnancy rates [68]. Balasch et al. showed that successful and unsuccessful implantation cycles did not differ in estradiol, progesterone and prolactin levels [69]. Moreover, hyperprolactinemia was present in 20% of the patients, but the conception rate and pregnancy outcome were similar in patients with normal prolactin levels and those having hyperprolactinemia [69]. Similar results were obtained by Oda et al. who studied the relationship between serum prolactin level three days before oocytes retrieval and IVF outcome in euprolactinemic patients with tubal infertility [70]. They found similar fertilization and cleavage rates in patients classified as having hyperprolactinemia (prolactin ≥ 30 μg/L) and those with normal prolactin levels. However, significantly lower fertilization rates were found in patients with prolactin < 10 μg/L compared with patients with hyperprolactinemia and lower cleavage rates were achieved in the low prolactin group in contrast with women with normal or elevated serum concentrations [70]. While most pregnancies were obtained in the euprolactinemic group, the sample size was too small to reach statistical significance [70]. Hofmann et al. found a similar number of oocytes and pregnancy rates in patients with transient hyperprolactinemia or without during gonadotropin-stimulated cycles for IVF [66]. Forman et al. also found no association of elevated plasma or follicular fluid prolactin concentration with oocytes or embryonic development and pregnancy occurrence in patients undergoing ovarian stimulation for IVF [67]. In the study of Gonen et al., no difference was noticed in fertilization rates and the number of oocytes retrieved according to prolactin level measured on the day after HCG administration [71].

On the other hand, a few studies showed that transitory hyperprolactinemia might have a detrimental effect on follicular or oocyte development [56,72]. Thus, Reinthaller et al. found lower fertilization rate in patients with transient hyperprolactinemia in comparison with patients without high prolactin levels during gonadotropin stimulation [72], the authors concluding that treatment of transient hyperprolactinemia might be beneficial for IVF outcome. Other authors demonstrated that untreated transient anaesthesia-induced hyperprolactinemia is associated with a lower proportion of fertilized oocytes advancing to cleaving embryos in patients undergoing IVF, although oocyte fertilization and pregnancy rate were unaffected [56]. Tarín et al. developed a predictive model for women’s assisted fecundity before starting the first IVF/ICSI treatment cycle which included prolactin as a prognostic factor which was negatively associated with IVF success [73].

However, based on other authors’ reports, it seems that a higher prolactin level might be positively associated with IVF outcomes. Thus, Zhang et al. showed that patients with better-cumulated pregnancy outcomes had higher prolactin levels and a profoundly increasing trend in prolactin levels during ovarian stimulation before IVF [74]. Moreover, transient elevations of prolactin during the IVF stimulation protocol in the range of more than 200% were found to be associated with an increase in the number of follicles with a mean diameter ≥ 12 millimetres and with more mature oocytes and better IVF success rate [75]. A study which aimed to evaluate the impact of serum prolactin concentration on the day of human chorionic gonadotrophin (HCG) administration of IVF/ICSI-embryo transfer procedures divided patients into groups based on prolactin values: group A values < 30 ng/mL, group B values between 30–60 ng/mL, group C values between 60–90 ng/mL and group D with values ≥ 90 ng/mL [8]. During COS serum prolactin levels increased significantly from baseline values leading to transient hyperprolactinemia [8]. In group A, the number of oocytes was the lowest, suggesting that this could be associated with low estrogen levels [8]. Authors hypothesized that prolactin may be indirectly associated with the number of oocytes obtained and that there might be a feedback loop between the estradiol secreted by the ovarian follicles and prolactin secretion from the pituitary gland [8]. The implantation rate and the pregnancy rate were the highest when prolactin levels were between 30–60 ng/mL [8]. Conversely, significantly lower implantation rates and pregnancy rates were found in group D compared to the other groups [8]. Another study evaluated the hormonal profiles in the mid-luteal phase of super-ovulated IVF cycles and found that patients with successful pregnancy had higher levels of prolactin than those with early pregnancy loss, suggesting a defect in endometrial decidualization associated with lower prolactin [71]. Moreover, the estradiol to prolactin ratio was highest in the group with early pregnancy loss and authors hypothesize that there was a poor response to serum estradiol and that adequate prolactin levels may be mandatory for maintaining a pregnancy after IVF-embryo transfer [76].

Due to better control of male factors such as poor sperm quality, ICSI success largely depends on oocyte quality. Several studies evaluated the relationship between serum levels of prolactin and ICSI outcomes. Kamel et al. found that, in ICSI cycles, transient hyperprolactinemia before ovum pickup was associated with higher-quality embryos [13]. However, no association with the clinical pregnancy rates was observed between prolactin levels in the mid-luteal phase, before and 2 h after the ovum pickup procedure and before embryo transfer [13]. The lack of a relationship between mid-luteal phase prolactin and pregnancy rate was also reported by other authors [77].

The relationship between prolactin level before IVF procedure and IVF outcome is largely unknown, very few studies evaluated this aspect, letting the clinician without a verified tool when facing a hyperprolactinemic patient undergoing IVF. Thus, Gonen et al. found no difference in serum prolactin levels before ovarian stimulation between pregnant and non-pregnant patients [78]. Moreover, Pattinson et al. found no relationship between the initial prolactin levels and the number of follicles, oocytes or pregnancies obtained [55]. Zhang et al. investigated the impact of hyperprolactinemia on the outcomes of IVF/ICSI and observed significantly higher numbers of oocytes and embryos in patients with basal prolactin levels higher than 16.05 ng/mL, but lower than 50 ng/mL [74]. Cumulative clinical pregnancy was positively related to prolactin concentrations between 30 and 50 ng/mL and levels higher than 40 ng/mL were associated with higher cumulative live birth rates [74].

### Prolactin Relation with Hyper-Response to COS and Ovarian Hyperstimulation Syndrome

Although clinical studies are lacking, the role of prolactin in mediating the impact of high estrogens on decidualization in high responders was highlighted by an experimental study treating human endometrial stromal cells with a decidualization cocktail with three different estradiol concentrations. The authors noticed that, although endometrial prolactin increased in all three regimens, prolactin was less with the high estradiol dose. Their conclusion was that supraphysiologic estradiol levels associated with high IVF responders may alter endometrial decidualization, implantation and placentation by affecting, among other mediators, the endometrial prolactin [79].

Ovarian hyperstimulation syndrome (OHSS) is the most severe and life-threatening complication of COS that is characterized by the occurrence of endothelial dysfunction, increases in vascular permeability and accumulation of fluid in the third space. In an attempt to identify the risk factors for the development of OHSS, a study evaluating 671 patients found that higher prolactin levels are linked to the development of OHSS in patients undergoing IVF and have been incorporated into a prediction model [80].

## 5. The Role of Dopamine Agonist Administration in IVF Treatment

It was shown that women with hyperprolactinemia, even with treatment, had lower parity, and older age at first pregnancy, although there appears to be no increased risk of pregnancy complications or adverse outcomes. [81] Thus, the possible impact of treated hyperprolactinemia on IVF outcome was evaluated in several studies, with divergent results. Therefore, Duan et al. [14] performed a retrospective study and found that the cumulative live birth rates, number of pregnancies and perinatal outcomes were similar between patients with treated hyperprolactinemia and those with normal prolactin levels undergoing IVF (Table 1) [14]. In patients with persistently elevated prolactin after treatment (median levels 88.35 ng/mL) the number of metaphase II oocytes was lower, but no other differences were observed between the groups [14]. Median prolactin levels before dopamine agonist treatment in the case group were 80 ng/mL and it reduced to around 15 ng/mL with treatment, similar to levels in the control group [14]. Doldi et al. divided patients with mild hyperprolactinemia before COS (mean values of serum prolactin in the study group of 24.7 ng/mL), undergoing ICSI, into two groups: one group received dopamine agonists, either cabergoline or bromocriptine, and the other group did not receive treatment. They found that in the group with untreated hyperprolactinemia a decreased FSH requirement, a higher number of good-quality oocytes (metaphase 2, mature oocytes), increased fertilization rate and higher numbers of embryos transferred were noticed (Table 1) [82].

Since the transient hyperprolactinemia occurring during COS was considered by some to negatively influence fertilization and/or implantation [72,83], the effect of treatment with dopamine agonists on IVF outcome was evaluated in a few studies (Table 1). Thus, administration of bromocriptine in patients with transient hyperprolactinemia in previous stimulated cycles was associated with higher fertilization rate in comparison with patients with untreated transient prolactin increase, but similar fertilization rates in patients without prolactin increase during ovarian stimulation [72]. Similarly, in unstimulated cycles of women with longstanding idiopathic infertility and with mild transitory elevations of prolactin in the range of 27–70 ng/mL lasting for 1–3 days and coinciding with preovulatory estradiol increases, bromocriptine administration was reported to be associated with higher conception rates [84]. However, other authors did not recommend dopamine agonist treatment in patients with transient hyperprolactinemia [78].

In patients with transient hyperprolactinemia during laparoscopy for oocyte collection, treatment with bromocriptine did result in a positive influence on embryonic development after IVF, although fertilization and pregnancy rates were similar (Table 1) [56]. On the other hand, another study examining the impact of anaesthesia-induced hyperprolactinemia and bromocriptine administration on IVF outcomes did not relate the change in prolactin concentrations with the occurrence of pregnancy [57].

Bromocriptine administration was studied in patients without hyperprolactinemia undergoing ovarian hyperstimulation for IVF, for prolactin suppression, but no impact was observed in terms of the number of oocytes, embryo quality and pregnancies in a double-blind placebo-controlled study [85]. However, it was noted that low prolactin concentration (median value of 5 μg/L) in association with higher estradiol levels during the follicular phase was correlated with an increase in placental protein 14, a glycoprotein which rises in the luteal phase, inhibits cell immune function and plays an essential role in the pregnancy process [85].

Bromocriptine administration was also studied in other situations assumed to be associated with a transitory increase in prolactin levels. Thus, normoprolactinemic clomiphene citrate-resistant women were suggested to have exaggerated prolactin response to TSH-releasing hormone and latent or intermittent hyperprolactinemia [86]. Thus, bromocriptine treatment was studied as adjunctive therapy in clomiphene-resistant patients with polycystic ovary syndrome and normal prolactin levels, but no significant effect was observed, except for the lowering of prolactin levels (Table 1) [86].

The role of prolactin in restoring the responsiveness of granulosa cells to stimulation was explored in an older study. Thus, Jinno et al. [87] proposed a new method of ovarian stimulation for IVF in patients with previous IVF failure, the bromocriptine rebound (BR) method [87]. They administrated bromocriptine from day 4 of the preceding cycle until 7 days before stimulation in patients treated with long agonist protocol and HMG. They reported that patients treated with the BR method had higher numbers of follicles, fertilized oocytes, embryos with superior morphology, clinical pregnancy and live birth rates in comparison with patients who did not receive bromocriptine (Table 1) [87]. Prolactin levels were also significantly higher in patients treated with bromocriptine and were correlated with the number of superior embryos [87]. They also noticed that the mRNA prolactin receptors in granulosa cells were higher in non-pregnant patients before bromocriptine administration and that the mRNA prolactin receptors decreased after BR method treatment. Thus, the authors concluded that IVF failure is the consequence of a granulosa cell resistance to prolactin action and that the bromocriptine-induced hypoprolactinemia contributes to the restoration of prolactin responsiveness and, subsequently, improved oocytes maturation [87]. A more recent study confirmed the results with the BR method [88]. Bromocriptine was administered daily from day 5 of the preceding cycle until 7 days before ovarian stimulation in women with normal prolactin levels and previous repeated unsuccessful ART in a new long agonist protocol cycle and the results were compared with those of women treated with long agonist protocol only [88]. The number of retrieved oocytes was the same in both groups; however, superior results were obtained with the BR method in terms of the number of fertilized oocytes, cleaved embryos, transplanted embryos, the ratio of good embryos, embryo score, and clinical pregnancy rates. The authors concluded that this method could be of value in attaining superior-quality embryos and transplantation rates in women with previous unsuccessful IVF/ICSI (Table 1) [88].

**Table 1:** Studies evaluating the efficacy of dopamine agonist administration on in vitro fertilization outcome.

Study	No of Patients	Type of the Study	Study Population	Type of HPRL	IVF Outcome	Prolactin Levels before Dopamine Agonist Treatment
Duan et al. (2019) [14]	535 pts	Retrospective	123 pts with treated HPRL and 369 matched controls	Preexistent to COS	CLBR similar in the two groups (69.1% versus s 66.4%, *p* = 0.58)	Median prolactin 80 ng/mL (study group) vs. 15.17 ng/mL (controls)
Doldi et al. (2000) [82]	135 pts	Prospective	59 pts with HPRL treated with BRC and 76 pts with HPRL without treatment	Preexistent to COS, mild HPRL in a cycle before COS	In the group with untreated HPRL a higher number of mature oocytes (87.9% versus 80.4%; *p* < 0.05), increased fertilization rate (70.8 +/− 28.0 versus 60.8 +/− 28.5; *p* < 0.03) and higher numbers of embryos transferred (3.6 +/− 1.6 versus 3.2 +/− 1.5; *p* < 0.05)	Mean 24.74 ± 3.17 ng/mL
Reinthaller et al. (1988) [72]	50 cycles, 40 pts	Prospective	18 pts with PRL ≤ 25 ng/mL (group 1), 15 pts with PRL > 25 ng/mL (group 2), 17 pts treated with BRC 3.75 mg/day (group 3)	Transient during COS	Higher fertilization rate (81.5%) in group 3 in comparison with group 2 (40.9%), but similar fertilization rates to group 1 (65.9%)	
Sopelak et al. (1989) [56]	32 pts	Prospective	19 controls and 13 pts treated with BRC 2.5 mg at 1 h (7 pts) and 12 h (6 pts) before anaesthesia	Transient, during anaesthesia for oocyte retrieval laparoscopy	95% cleaving embryo in treated pts vs. 63% in controls, *p* < 0.001, similar fertilization and pregnancy rates	22.4 ± 5.2 ng/mL in controls, 7 ± 1.7 ng/mL (1 h), 3.1 ± 0.3 ng/mL (12 h)
Taylor et al. (1986) [57]	20 pts	Prospective	10 pts treated with BRC, 10 pts without treatment	Transient, during anaesthesia for oocyte retrieval laparoscopy	No relationship between change in PRL and occurence of pregnancy	
Seppälä et al. (1989) [85]	35 pts	Double-blind placebo-controlled study	17 pts treated with BRC 1.25 mg/day from day 2 till HCG administration 18 pts treated with placebo	Normal PRL levels	Number of oocytes, embryo quality and pregnancies were similar between groups	
Parsanezhad et al. (2004) [86]	100 pts	Prospective, double-blind, placebo-controlled study	CC-resistant normoprolactinemic PCOS women 47 pts treated with CC 200 mg × 5 days plus BRC 7.5 mg/day and 53 pts with placebo	Normal PRL levels	Similar ovulation rate in treated vs. placebo	
Jinno et al. (1997) [87]	162 cycles	Prospective randomized study	Ovulatory women with previous IVF failure (82 cycles treated with BRC rebound method and 80 cycles with long protocol)	Normal PRL levels	Clinical pregnancy and live birth rates were higher (38% and 33%) in BRC rebound group versus long protocol (21% and 19%)	
Moride et al. (2018) [88]	121 pts	Retrospective	Pts with repeated unsuccessful ART (22 women treated with BRC rebound method and 99 pts with long protocol)	Normal PRL levels	superior results were obtained with the BR method in terms of higher number of fertilized oocytes, cleaved embryos, transplanted embryos and clinical pregnancy rates (27.2% vs. 10.1%) in BRC rebound group	5.9 ± 3.3 ng/mL (BRC rebound) vs. 7.5 ± 8.3 ng/mL (long agonist)

HPRL: hyperprolactinemia; Pts: patients; IVF: in vitro fertilization; COS: controlled ovarian stimulation; CLBR: cumulative live birth rate; BRC: bromocriptine; PRL: prolactin; HCG: human chorionic gonadotropin; PCOS: polycystic ovary syndrome; CC: clomiphene citrate; ART: assisted reproductive technologies.

## 6. Conclusions and Future Directions

Although an increasing body of evidence supports the involvement of prolactin in the physiology and pathology of reproduction by several mechanisms, the data available in the literature regarding the optimal level of circulating prolactin for the best IVF treatment outcome are scarce. Hyperprolactinemia is a well-known cause of infertility throiugh an inhibitory effect on gonadotropin production. However, other mechanisms may also be involved such as endometrial dysfunction and immune dysregulation. On the other hand, prolactin may be involved in the physiology of reproduction. Thus, although not all studies agree, there are data supporting that higher levels of prolactin in follicular fluid are associated with increased oocyte competence. It is also possible that prolactin contributes to corpus luteum formation and survival, endometrial receptivity, blastocyst implantation potential and survival of low-motile sperm. During IVF, variations in prolactin levels were reported, characterized as transient hyperprolactinemia, possible causes being gonadotropins and GnRH agonists administration, increased estradiol levels and procedure-related stress. The association of transient hyperprolactinemia with IVF results was variably reported by different studies, most of the studies failing to demonstrate a relationship. However, a limited number of studies showed that higher prolactin levels during IVF are associated with higher implantation and pregnancy rates, and better-cumulated pregnancy outcomes. It is possible that the moment of the measurement of prolactin level is a significant factor in explaining the differences in the results. Regarding the benefits of treatment of hyperprolactinemia preceding IVF, the data are scarce and divergent. It seems that treatment of hyperprolactinemia does not negatively impact the pregnancy rate, although the effect of persistently high prolactin levels on oocyte quality is controversial. Treatment of transient hyperprolactinemia during IVF or spontaneous cycles was suggested to be beneficial in terms of fertilization rates and conception rates, although a limited amount of evidence is available. Administration of bromocriptine followed by withdrawal of treatment might be successful for obtaining a pregnancy in previous IVF failure patients by restoring granulosa cells’ responsiveness to prolactin.

Future studies are necessary to clarify apparently divergent laboratory and clinical data and to establish the optimal serum prolactin level for the best IVF outcome in patients with hyperprolactinemia preceding the IVF treatment and those with transient hyperprolactinemia during IVF.

## Data Availability

Not applicable.

## Abbreviations

ART: assisted reproductive techniques; BR, bromocriptine rebound; COS, controlled ovarian stimulation; GnRH, gonadotrophin-releasing hormone; FSH, follicle-stimulating hormone; HCG, human chorionic gonadotrophin; HMG, human menopausal gonadotrophin; ICSI, intracytoplasmic sperm injection; IL-1, interleukin 1; IVF, in vitro fertilization; LH, luteinizing hormone; mRNA, messenger ribonucleic acid; OHSS, ovarian hyperstimulation syndrome; RIF, repeated implantation failure.
